# A conceptual DFT study of the molecular properties of glycating carbonyl compounds

**DOI:** 10.1186/s13065-017-0239-7

**Published:** 2017-01-23

**Authors:** Juan Frau, Daniel Glossman-Mitnik

**Affiliations:** 1 Departament de Química, Universitat de les Illes Balears, Carretera de Valldemossa, Km 7.5, 07010 Palma, Spain; 2 Departamento de Medio Ambiente y Energía, Laboratorio Virtual NANOCOSMOS, Centro de Investigación en Materiales Avanzados, Miguel de Cervantes 120, Complejo Industrial Chihuahua, 31136 Chihuahua, Chih Mexico

**Keywords:** Computational chemistry, Molecular modeling, Glycating carbonyl compounds, Maillard reaction, Conceptual DFT, Chemical reactivity theory

## Abstract

Several glycating carbonyl compounds have been studied by resorting to the latest Minnesota family of density functional with the objective of determinating their molecular properties. In particular, the chemical reactivity descriptors that arise from conceptual density functional theory and chemical reactivity theory have been calculated through a $\Delta$SCF protocol. The validity of the KID (Koopmans’ in DFT) procedure has been checked by comparing the reactivity descriptors obtained from the values of the HOMO and LUMO with those calculated through vertical energy values. The reactivity sites have been determined by means of the calculation of the Fukui function indices, the condensed dual descriptor $\Delta {f}(\mathbf {r})$ and the electrophilic and nucleophilic Parr functions. The glycating power of the studied compounds have been compared with the same property for simple carbohydrates.

**Electronic supplementary material:**

The online version of this article (doi:10.1186/s13065-017-0239-7) contains supplementary material, which is available to authorized users.

**Graphical abstract:**
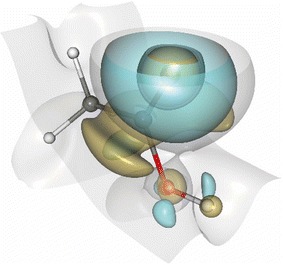
Several glycating carbonyl compounds have been studied by resorting to the latest Minnesota family of density functional with the objective of determinating their molecular properties, the chemical reactivity descriptors and the validity of the KID (Koopmans’ in DFT) procedure

## Introduction

It is already well known that several diseases like diabetes, Alzheimer and Parkinson are related to the formation of the so called advanced glycation endproducts (AGEs). These toxic molecules are the result of a chain of reactions that is initiated by a nucleophilic addition between a reducing carbonyl compound and the amino groups of amino acids, peptides, and proteins. This is a nonenzymatic reaction (nonenzymatic glycation or Maillard reaction) that leads to the formation of a freely reversible Schiff base. Glycated amino acids and proteins can undergo further reactions, giving rise to the AGEs [1].

Thus, it is very important to understand how the different molecules bearing a reducing carbonyl group react with the amino acids and proteins and to obtain a measure of the extent of this reaction in each case. The glycating power, that is, the abilty of different molecules with reducing carbonyl groups to interact with the amino group of a proteins is strongly dependent on their molecular structures and electronic properties. This knowledge could be of interest for the design of new therapeutic drugs and AGEs inhibitors.

In a very interesting work, Adrover et al. [2] have studied the kinetics of the interaction of some potential inhibitors of the formation of AGEs with various glycating carbonyl compounds. They found that the rate constants for the initial reaction between the carbonyl group of each glycating compound with the amine group of pyridoxamine are strongly dependent on their molecular structures.

In a previous work, we have found that the glycation power of simple carbohydrates can be quantified in terms of the electronic properties of such molecules. In particular, it has been proved that good correlations exist between the glycation power and some descriptors that arise from conceptual density functional theory (DFT). This theory, or chemical reactivity theory (as it is also known) is a powerful tool for the prediction, analysis and interpretation of the outcome of chemical reactions [3–6].

From an empirical and practical point of view, it meaningful to follow the procedure of assigning the KS HOMO as equal to and opposite of the vertical ionization potential, $\epsilon _H$ = −I and the KS LUMO as equal to and opposite of the vertical electron affinity, $\epsilon _L$ = −A. We have coined the acronym KID for this empirical procedure (for “Koopmans in DFT”). This means that how well a given density functional behaves can be estimated by checking how well it follows the “Koopmans in DFT” (KID) procedure and this will be crucial for a good calculation of the Conceptual DFT descriptors that predict and explain the chemical reactivity of molecular systems. However, we have already observed that this is fulfilled with varying accuracy for different approximate density functionals and molecular systems [7–13].

This means that the goodness of a given density functional that allows to predict and explain the chemical reactivity of a molecular system can be estimated by checking how well it follows the KID procedure. Thus, it is interesting to study the performance of some new density functionals that have shown great accuracy across a broad spectrum of databases in chemistry and physics [14] on the fulfilling of the KID procedure because only well-behaved density functionals should be used for the calculation of molecular properties.

**Fig. 1:**
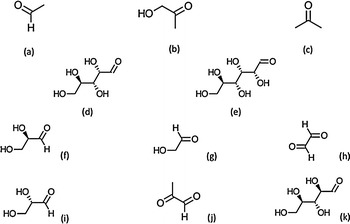
Molecular structures of** a** Acetaldehyde,** b** Acetol,** c** Acetone,** d** Arabinose,** e** Glucose,** f** d-glyceraldehyde,** g** Glycoladehyde,** h** Glyoxal,** i** l-glyceraldehyde,** j** Methylglyoxal and** k** Ribose

The objective of this work is twofold: (i) to conduct a comparative study of the performance of several of the latest Minnesota family of density functionals for the description of the chemical reactivity of some glycating carbonyl compounds which molecular structures are shown in Fig. 1; and (ii) to perform a comparison of the glycation power by relating the experimental rate constants for the initial reaction (or Maillard) of those molecules with amino groups, with accurately calculated Conceptual DFT descriptors.

## Theoretical background

As this work is part of an ongoing project, the theoretical background related to the conceptual DFT global descriptors is similar to that presented in previous research and has been already described in detail before [7–13].

For the case of the conceptual DFT local descriptors, it is worth to mention that the Fukui function is defined in terms of the derivative of $\rho (\mathbf {r})$ with respect to *N* and reflects the ability of a molecular site to accept or donate electrons so two definitions of the Fukui function do exist. The first one, $f^{+}(\mathbf {r})$, has been associated to reactivity for a nucleophilic attack so that it measures the intramolecular reactivity at the site $\mathbf {r}$ towards a nucleophilic reagent. The second one, $f^{-}(\mathbf {r})$, has been associated to reactivity for an electrophilic attack so that this function measures the intramolecular reactivity at the site $\mathbf {r}$ towards an electrophilic reagent [15].

Morell et al. [5, 16–21] have proposed a local reactivity descriptor (LRD) which is called the dual descriptor (DD) $f^{(2)}(\mathbf {r})\equiv {\Delta }f(\mathbf {r})$. The dual descriptor can be condensed over the atomic sites: when $\Delta$f$_{k}>0$ the process is driven by a nucleophilic attack on atom *k* and then that atom acts as an electrophilic species; conversely, when $\Delta$f$_{k}<0$ the process is driven by an electrophilic attack over atom *k* and therefore atom *k* acts as a nucleophilic species.

In 2014, Domingo proposed the nucleophilic and electrophilic Parr functions P($\mathbf r$) [22, 23] as an alternative to the Fukui functions: $P{^-}(\mathbf {r}) = \rho _{s}^{rc} (\mathbf r)$ (for electrophilic attacks) and $P{^+}(\mathbf {r}) = \rho _{s}^{ra} (\mathbf r)$ (for nucleophilic attacks) which are related to the atomic spin density (ASD) at the **r** atom of the radical cation or anion of a given molecule, respectively. The ASD over each atom of the radical cation and radical anion of the molecule gives the local nucleophilic P$^-_k$ and electrophilic P$^+_k$ Parr functions of the neutral molecule [24].

Another local reactivity descriptor has been defined so that it permits to measure local reactivities according to the molecular size [18, 19]. Such a descriptor is the local hypersoftness (LHS) whose working equation is expressed as follows: $LHS \approx \Delta f(\mathbf {r})\cdot S^{2}$ where *S* stands for the global softness [3, 25, 26]. As the local hypersoftness can be condensed over the atomic sites, the condensed local hypersoftness is simply computed as $LHS \simeq \left( f_{k}^{+} - f_{k}^{-}\right) \cdot \left( \epsilon _L- \epsilon _H\right) ^{-2}$. The procedure is explained as follows: $f^{(2)}_{k}$ is expressed in atomic units, meanwhile *S* is measured in mili eV raised to the power of −1, however before performing the multiplication, the mili factor is turned back into 10^−3^ and then *S* is raised to the power of 2; the resulting value uses the unit mili eV raised to the power of −2, meaning m (eV^−2^); the parenthesis is put in order to make clear that the prefix mili is not raised to the power of −2.

## Setting and computational methods

Following our previous work [7–13], all computational studies were performed with the Gaussian 09 [27] series of programs with density functional methods as implemented in the computational package. The equilibrium geometries of the molecules were determined by means of the gradient technique. The force constants and vibrational frequencies were determined by computing analytical frequencies on the stationary points obtained after the optimization to check if there were true minima. The basis set used in this work was Def2SVP for geometry optimization and frequencies while Def2TZVP was considered for the calculation of the electronic properties [28, 29].

For the calculation of the molecular structure and properties of the studied systems, we have chosen several density functionals from the latest Minnesota density functionals family, which consistently provide satisfactory results for several structural and thermodynamic properties [14]: M11, which is a is a range-separated hybrid meta-GGA [30], M11L, which is a dual-range local meta-GGA [31], MN12L, which is a nonseparable local meta-NGA [32], MN12SX, which is a range-separated hybrid nonseparable meta-NGA [33], N12, which is a nonseparable gradient approximation [34], N12SX, which is a range-separated hybrid nonseparable gradient approximation [33], SOGGA11, which is a GGA density functional [35] and SOGGA11X, which is a hybrid GGA density functional [36]. In these functionals, GGA stands for generalized gradient approximation (in which the density functional depends on the up and down spin densities and their reduced gradient) and NGA stands for nonseparable gradient approximation (in which the density functional depends on the up/down spin densities and their reduced gradient, and also adopts a nonseparable form). All the calculations were performed in the presence of water as a solvent, by doing IEF-PCM computations according to the SMD solvation model [37].

## Results and discussion

### Global descriptors

The molecular structures of acetaldehyde, acetol, acetone, arabinose, glucose, d-glyceraldehyde, glycoladehyde, glyoxal, l-glyceraldehyde, methylglyoxal, ribose and N1DDFLT were pre-optimized by starting with the readily available MOL structures (ChemSpider: http://www.chemspider.com, PubChem: pubchem.ncbi.nlm.nih.gov), and finding the most stable conformers by means of the Avogadro 1.2.0 program [38, 39] through a random sampling with molecular mechanics techniques and a consideration of all the torsional angles through the general AMBER force field [40]. The structures of the resulting conformers were then reoptimized with the eight density functionals mentioned in the previous section in conjunction with the Def2SVP basis set and the SMD solvation model, using water as a solvent.

As the validity of the KID procedure could be controversial, we have started with the calculation of the conceptual DFT global descriptors: global electronegativity $\chi$, the global hardness $\eta$ and the global electrophilicity $\omega$ for the studied systems, both through a $\Delta$SCF procedure and wlth the values of the orbital energies from the HOMO and LUMO. We have extended the calculations in order to include the electrodonating ($\omega ^{-}$) and electroaccepting ($\omega ^{+}$) powers as well as the net electrophilicity $\Delta \omega ^{\pm }$ for further verifications.

The HOMO and LUMO orbital energies (in eV), ionization potentials I and electron affinities A (in eV), and global electronegativity $\chi$, total hardness $\eta$, global electrophilicity $\omega$, electrodonating power, ($\omega ^{-}$), electroaccepting power ($\omega ^{+}$), and net electrophilicity $\Delta \omega ^{\pm }$ of the studied glycating carbonyl compounds calculated with the eight density functionals and the Def2TZVP basis set using water as as solvent simulated with the SMD parametrization of the IEF-PCM model are presented in Additional file 1: Tables S1A–S8A. The upper part of the tables shows the results derived assuming the validity of the KID procedure (hence the subscript K) and the lower part shows the results derived from the calculated vertical I and A. It should be remembered that only the vertical energy differences must be included instead of the adiabatic ones, because the Conceptual DFT descriptors have been defined at a constant external potential v($\mathbf {r}$).

With the object of analyzing our results and in order to check for the assessment of the KID procedure, we have previously designed several accuracy descriptors (AD) that relate the results obtained through the HOMO and LUMO calculations with those obtained by means of the vertical I and A within a $\Delta$SCF procedure. The first three AD are related to the simplest fulfillment of the KID procedure by relating $\epsilon _H$ with −I, $\epsilon _L$ with −A, and the behavior of them in the description of the HOMO-LUMO gap: $J_{I} = |\epsilon _H + E_{gs} (N-1) - E_{gs} (N)|$, $J_{A} = |\epsilon _L + E_{gs} (N) - E_{gs} (N+1)|$ and $J_{HL} = \sqrt{{J_{I}}^2 + {J_{A}}^2}$. Next, we consider four other descriptors that analyze how well the studied density functionals are useful for the prediction of the electronegativity $\chi$, the global hardness $\eta$ and the global electrophilicity $\omega$, and for a combination of these Conceptual DFT descriptors, just considering the energies of the HOMO and LUMO or the vertical I and A: $J_{\chi } = |\chi - \chi _K| ,\quad J_{\eta } = |\eta - \eta _K|, \quad J_{\omega } = |\omega - \omega _K|$ and $J_{D1} = \sqrt{{J_{\chi }^2} + {J_{\eta }^2} + {J_{\omega }^2}}$, where D1 stands for the first group of conceptual DFT descriptors. Finally, we designed other four AD to verify the goodness of the studied density functionals for the prediction of the electroaccepting power ($\omega ^{+}$), the electrodonating power ($\omega ^{-}$), the net electrophilicity $\Delta \omega ^{\pm }$, and for a combination of these Conceptual DFT descriptors, just considering the energies of the HOMO and LUMO or the vertical I and A: $J_{\omega ^{+}} = |\omega ^{+} - \omega ^{+}_K| , \quad J_{\omega ^{-}} = |\omega ^{-} - \omega ^{-}_K| , \quad J_{\Delta \omega ^{\pm }} = |\Delta \omega ^{\pm } - \Delta \omega ^{\pm }_K|$ and $J_{D2} = \sqrt{{J_{\omega ^{-}}^2} + {J_{\omega ^{+}}^2} + {J_{\Delta \omega ^{\pm }}^2}}$, where D2 stands for the second group of Conceptual DFT descriptors.

The results of the calculations of J_*I*_, J_*A*_, J_*HL*_, $J_{\chi }$, $J_{\eta }$, $J_{\omega }$, $J_{D1}$, $J_{\omega ^{+}}$, $J_{\omega ^{-}}$, $J_{\Delta \omega ^{\pm }}$ and $J_{D2}$ for the glycating carbonyl compounds considered in this work are displayed in Additional file 1: Tables S1B–S8B.

On the basis of the results for the descriptors presented on Additional file 1: Tables S1B–S8B, we have compiled the average values for for each density functional on the whole group of glycating carbonyl compounds, and the calculated results are displayed on Table 1.

**Table 1:** Average descriptors J_*I*_, J_*A*_, J_*HL*_, $J_{\chi }$, $J_{\eta }$, $J_{\omega }$, $J_{D1}$, $J_{\omega ^{+}}$, $J_{\omega ^{-}}$, $J_{\Delta \omega ^{\pm }}$ and $J_{D2}$  for the acetaldehyde, acetol, acetone, arabinose, glucose, d-glyceraldehyde, glycolaldehyde, glyoxal, l-glyceraldehyde, methylglyoxal and ribose molecules calculated with the M11, M11L, MN12L, MN12SX, N12, N12SX, SOGGA11 and SOGGA11X density functionals and the Def2TZVP basis set using water as as solvent simulated with the SMD parametrization of the IEF-PCM model

	J_*I*_	J_*A*_	J_*HL*_	$J_{\chi }$	$J_{\eta }$	$J_{\omega }$	$J_{D1}$	$J_{\omega ^{-}}$	$J_{\omega ^{+}}$	$J_{\Delta \omega ^{\pm }}$	$J_{D2}$
M11	2.72	2.83	3.93	0.08	5.55	1.01	5.66	1.70	1.65	3.35	4.11
M11L	0.46	0.30	0.56	0.08	0.77	0.31	0.86	0.53	0.61	1.14	1.40
MN12L	0.37	0.26	0.46	0.06	0.63	0.22	0.69	0.37	0.43	0.80	0.98
MN12SX	0.17	0.18	0.26	0.04	0.35	0.11	0.37	0.19	0.19	0.38	0.47
N12	0.65	0.67	0.94	0.08	1.32	0.72	1.56	1.35	1.34	2.70	3.31
N12SX	0.05	0.14	0.15	0.05	0.17	0.09	0.21	0.19	0.14	0.33	0.41
SOGGA11	0.72	1.12	1.40	0.31	1.84	1.00	2.22	1.98	1.79	3.77	4.63
SOGGA11X	1.24	1.21	1.73	0.05	2.45	0.58	2.53	1.00	1.01	2.01	2.46

As can be seen from the results on Table 1, the KID procedure holds with great accuracy for the MN12SX and N12SX density functionals, which are range-separated hybrid meta-NGA and range-separated hybrid NGA density functionals, respectively. It must be stressed that it was not our intention to perform a gap-fitting by minimizing a descriptor by choosing an optimal range-separation parameter, but to check if the density functionals considered in this study fulfill the KID procedure. Indeed, the values of J_*I*_, J_*A*_ and J_*HL*_ are not exactly zero. However, their values can be favorably compared with the results presented for these quantities in the work of Lima et al. [41], where the minima has been obtained by choosing a parameter that enforces that behavior.

It is interesting to see that the same density functionals also fulfill the KID procedure for the other descriptors, namely $J_\chi$, $J_\eta$, $J_\omega$, and $J_{D1}$, as well as for $J_{\omega ^{-}}$, $J_{\omega ^{+}}$, $J_{\Delta \omega ^{\pm }}$, and $J_{D2}$. These results are very important, because they show that it is not enough to rely only in J_*I*_, J_*A*_ and J_*HL*_. For example, if we consider only $J_\chi$, for all of the density functionals considered, the values are very close to zero. As for the other descriptors, only the MN12SX and N12SX density functionals show this behavior. That means that the results for $J_\chi$ are due to a fortuitous cancellation of errors.

The usual GGA (SOGGA11) and hybrid-GGA (SOGGA11X) are not good for the fulfillment of the KID procedure, and the same conclusion is valid for the local functionals M11L, MN12L and N12. An important fact is that although the range-separated hybrid NGA and range-separated hybrid meta-NGA density functionals can be useful for the calculation of the conceptual DFT descriptors, it is not the same for the range-separated hybrid GGA (M11) density functional. An inspection of Additional file 1: Table S1A shows that this is due to the fact that this functional describes inadequately the energy of the LUMO, leading to positive values of A (with the exception of glyoxal and methylglyoxal), which are in contradiction with the $\Delta$SCF results.

### Local descriptors

The condensed Fukui functions can also be employed to determine the reactivity of each atom in the molecule and have been calculated using the AOMix molecular analysis program [42, 43] starting from single-point energy calculations, while the condensed dual descriptor was calculated as $\Delta \text{f}_{k} = \text{f}_{k}^{+} - \text{f}_{k}^{-}$ [16, 17]. From the interpretation given to the Fukui function, one can note that the sign of the dual descriptor is very important to characterize the reactivity of a site within a molecule towards a nucleophilic or an electrophilic attack. That is, if $\Delta \text{f}_{k} > 0$, then the site is favored for a nucleophilic attack, whereas if $\Delta \text{f}_{k} < 0$, then the site may be favored for an electrophilic attack [16, 17, 44]. These results may be compared with the values of the electrophilic Parr function over the carbonyl C atoms of the studied compounds by means of the ASD of the corresponding radical anion.

The condensed Fukui functions, the condensed dual descriptor $\Delta \text{f}_{k}$ and the electrophilic $\text{P}^+_k$ Parr functions over the carbonyl C atoms of the acetaldehyde, acetol, acetone, arabinose, glucose, d-glyceraldehyde, glycoladehyde, glyoxal, l-glyceraldehyde, methylglyoxal and ribose molecules calculated with the MN12SX and N12SX density functionals and the Def2TZVP basis set using water as as solvent simulated with the SMD parametrization of the IEF-PCM model are shown in Table 2. For the calculation of the ASD, we have considered both a Mulliken Population Analysis (MPA) [45–48] or a Hirshfeld Population Analysis (HSA) [49–51] modified to render CM5 atomic charges [52].

**Table 2:** Electrophilic Fukui functions, condensed dual descriptors and electrophilic Parr functions for the acetaldehyde, acetol, acetone, arabinose, glucose, d-glyceraldehyde, glyoxal, glycolaldehyde, l-glyceraldehyde, methylglyoxal and ribose molecules calculated with the MN12SX and N12SX density functionals and the Def2TZVP basis set using water as as solvent simulated with the SMD parametrization of the IEF-PCM model

	MN12SX				N12SX			
	$\text{f}^{+}_{k}$	$\Delta \text{f}_k$	$\text{P}^+_k ({\textsc{mpa}})$	$\text{P}^+_k ({\textsc{hpa}})$	$\text{f}^{+}_{k}$	$\Delta \text{f}_k$	$\text{P}^+_k ({\textsc{mpa}})$	$\text{P}^+_k ({\textsc{hpa}})$
Acetaldehyde	0.67	0.57	0.76	0.56	0.66	0.56	0.72	0.57
Acetol	0.53	0.43	0.75	0.47	0.56	0.46	0.66	0.49
Acetone	0.54	0.45	0.75	0.47	0.56	0.48	0.67	0.48
Arabinose	0.63	0.56	0.72	0.53	0.63	0.54	0.67	0.54
Glucose	0.63	0.61	0.73	0.55	0.63	0.61	0.68	0.56
d-Glyceraldehyde	0.66	0.57	0.74	0.54	0.64	0.55	0.69	0.54
Glycolaldehyde	0.62	0.51	0.71	0.57	0.62	0.52	0.68	0.57
Glyoxal	0.60	0.38	0.52	0.52	0.58	0.38	0.52	0.52
l-Glyceraldehyde	0.66	0.57	0.74	0.54	0.64	0.55	0.69	0.55
Methylglyoxal	0.60	0.37	0.58	0.53	0.59	0.38	0.56	0.53
Ribose	0.62	0.58	0.72	0.54	0.62	0.59	0.68	0.55

*MPA* Mulliken population analysis,* HPA* Hirshfeld population analysis

### Glycating power

In a previous work [53], we have studied the glycating power (GP) of simple carbohydrates and tried to explain it in terms of the calculated conceptual DFT descriptors. To this end, we performed a Linear Regression Analysis (LRA) of the results of plotting the rate of condensation of monosaccharides with pyridoxamine (k_3_) [54] against the global electrophilicity $\omega$. A good relationship between the glycating power (GP) and the global electrophilicity $\omega$ was obtained for the model chemistry MN12SX/Def2TZVP/SMD(H2O), according to the following equation: GP = a × $\omega$ + b, where GP = k_3_, a is the slope and b is the interception of the linear correlation. The values of a and b were 87.5200 and −134.3312 respectively, giving rise to a MAD of 0.5840.

It could be interesting to perform a similar analysis for the glycating carbonyl compounds studied in this work starting from the values for the rate constants k$_1$ compiled by Adrover et al. [2]. The experimental values of k_1_ (in M^−1^ h^−1^) (taken from the mentioned work [2]) are reproduced here for the sake of convenience: Acetone = 3.9 × 10^1^, Acetol = 8.5 × 10^1^, Acetaldehyde = 3.0 × 10^4^, Glycolaldehyde = 2.2 × 10^5^, Glucose = 3.7 × 10^5^, Ribose = 3.9 × 10^5^, Arabinose = 2.9 × 10^5^, Glyoxal = 1.8 × 10^7^, Methylglyoxal = 1.1 × 10^6^. However, this is not an easy task because the k_1_ values for glyoxal and methylglyoxal are one or two orders of magnitude larger than for the other aldehydes (including aldoses) and several orders of magnitude larger than the ketones (acetol and acetone). This makes impossible to span accurately all the values within a LRA.

However, a qualitative trend may be observed in terms of the global electrophilicty $\omega$. An inspection of Additional file 1: Tables S4A–S6A of the ESI reveals that for MN12SX and N12SX density functionals, the results for glyoxal and methylglyoxal are larger than for the other molecules considered in this work, in agreement with the experimental results [2]. In turn, the values for acetol and acetone are the smallest ones, again in a good agreement with the experiments.

One could also expect that a similar trend could be obtained from the local descriptors presented in Table 2. Indeed, this is not case for the electrophilic Fukui function f$^{+}_{k}$ and the condensed dual descriptor $\Delta$ $\text{f}_{k}$ because the are sub-intensive properties. Now paying attention to the electrophilic Parr functions $\text{P}^+_k ({\textsc{mpa}})$ and $\text{P}^+_k ({\textsc{hpa}})$, it can be observed that there are no significative differences for the results in the first case, while the second predicts lower values for acetol and acetone, as it should be expected. However, this method fails to predict greater values for glyoxal and methylglyoxal.

It is worth to look at the results for d- and l-glyceraldehyde because they were not included in the experimental work of Adrover et al. [2]. Our calculations predict that the glycating power GP of both molecules will be slighty lower than the value for glucose.

The condensed local hypersoftness (LHS) over the carbonyl C atoms of the acetaldehyde, acetol, acetone, arabinose, glucose, d-glyceraldehyde, glycoladehyde, glyoxal, l-glyceraldehyde, methylglyoxal and ribose molecules calculated with the MN12SX and N12SX density functionals and the Def2TZVP basis set using water as as solvent simulated with the SMD parametrization of the IEF-PCM model are shown in Table 3.

**Table 3:** Condensed local hypersoftness (LHS) over the carbonyl C atoms of the acetaldehyde, acetol, acetone, arabinose, glucose,  d-glyceraldehyde, glyoxal, glycolaldehyde,  l-glyceraldehyde, methylglyoxal and ribose molecules calculated with the M06 and MN12SX density functionals and the Def2TZVP basis set using water as as solvent simulated with the SMD parametrization of the IEF-PCM model

	MN12SX	N12SX
Acetaldehyde	13.17	14.43
Acetol	11.04	12.82
Acetone	10.24	12.06
Arabinose	15.25	15.94
Glucose	17.82	19.80
d-Glyceraldehyde	13.79	14.73
Glycolaldehyde	12.61	14.07
Glyoxal	19.90	23.28
l-Glyceraldehyde	13.79	14.73
Methylglyoxal	20.20	22.06
Ribose	17.73	18.16

The results are noteworthy. If we take the LHS as a measure of the glycating power GP, it can be observed that for the MN12SX and N12SX density functionals, the values for glyoxal and methylglyoxal almost double those for the ketones (acetol and acetone). The other aldehydes (including the aldoses) display intermediate values. This is in agreement with the experimental results. Notwithstanding, there is a small discrepancy between both functionals. While MN12SX predicts that the GP of methylglyoxal will be (slighty) larger than that of glyoxal, only the second, N12SX, shows the correct trend, that is, GP (glyoxal) > GP (methylglyoxal).

## Conclusions

The Minnesota family of density functionals (M11, M11L, MN12L, MN12SX, N12, N12SX, SOGGA11 and SOGGA11X) have been tested for the fulfillment of the KID procedure by comparison of the HOMO- and LUMO-derived values with those obtained through a $\Delta$SCF procedure. It has been shown that the range-separated hybrid meta-NGA density functional (MN12SX) and the range-separated hybrid NGA density functional (N12SX) are the best for the accomplishment of this objective. As such, they represent a good prospect for their usefulness in the description of the chemical reactivity of molecular systems of large size.

From the whole of the results presented in this work, it can be seen that the sites of interaction of the glycationg carbonyl compounds can be predicted by using DFT-based reactivity descriptors such as the electronegativity, global hardness, global electrophilicity, electrodonating and electroaccepting powers, net electrophilicity as well as Fukui function, condensed dual descriptor and condensed local hypersoftness calculations. These descriptors were used in the characterization and successfully description of the preferred reactive sites and provide a firm explanation for the reactivity of those molecules.

Moreover, the difference in the glycating power GP between aldehydes and ketones could be explained in terms of the conceptual DFT descriptors. This is based on calculations performed with the MN12SX density functional in conexion with the Def2TZVP basis set and the SMD parametrization of the IEF-PCM model using water as a solvent. It can be concluded that this model chemistry [MN12SX/Def2TZVP/SMD (Water)] is the best for fulfilling the KID procedure and for the prediction of the glycating power GP of the carbonyl compounds and could be used for the study of the behavior of larger molecules bearing carbonyl C atoms capable of taking part in the Maillard reaction.

## Additional file

**Additional file 1.** Additional tables.
