# Supplementary material for: A conceptual DFT study of the molecular properties of glycating carbonyl compounds
Source: Chem Cent J. 2017 Jan 23;11:8. doi: 10.1186/s13065-017-0239-7 (PMC5256638; doi:10.1186/s13065-017-0239-7)
Supplement: Supplementary file 1 — Additional file 1. Additional tables. [file 13065_2017_239_MOESM1_ESM.pdf]

A Conceptual DFT Study of the Molecular Properties of Glycating Carbonyl Compounds

Electronic Supporting Information

Juan Frau and Daniel Glossman-Mitnik

Table S1A: HOMO and LUMO orbital energies (in eV), ionization potentials I and electron affinities A (in eV), and global electronegativity χ, total hardness η, global electrophilicity ω, electrodonating power (ω^-), electroaccepting power (ω^+), and net electrophilicity $\Delta\omega^\pm$ of Acetaldehyde, Acetol, Acetone, Arabinose, Glucose, d-Glyceraldehyde, Glyoxal, l-Glyceraldehyde, Methylglyoxal and Ribose calculated with the M11 density functional and the Def2TZVP basis set using water as solvent simulated with the SMD parametrization of the IEF-PCM model. The upper part of the table shows the results derived assuming the validity of KID procedure and the lower part shows the results derived from the calculated vertical I and A.

Property	HOMO	LUMO	χ_K	η_K	ω_K	ω_K^-	ω_K^+	$\Delta\omega_K^\pm$
Acetaldehyde	-10.35	1.61	4.37	11.95	0.80	4.53	0.16	4.69
Acetol	-9.97	1.63	4.17	11.60	0.75	4.31	0.14	4.45
Acetone	-10.18	1.79	4.20	11.97	0.74	4.32	0.12	4.44
Arabinose	-10.11	1.31	4.40	11.42	0.85	4.61	0.21	4.82
Glucose	-9.98	1.19	4.40	11.17	0.87	4.63	0.23	4.86
d-Glyceraldehyde	-10.49	1.33	4.58	11.82	0.89	4.80	0.22	5.02
Glycolaldehyde	-10.47	1.31	4.58	11.78	0.89	4.81	0.23	5.04
Glyoxal	-10.33	-0.62	5.47	9.71	1.54	6.43	0.95	7.38
l-Glyceraldehyde	-10.49	1.33	4.58	11.82	0.89	4.80	0.22	5.02
Methylglyoxal	-10.20	-0.40	5.30	9.79	1.43	6.13	0.83	6.96
Ribose	-10.16	1.15	4.50	11.31	0.90	4.75	0.25	5.00
**Property**	**I**	**A**	χ	η	ω	ω^-	ω^+	$\Delta\omega^\pm$
Acetaldehyde	7.39	1.31	4.35	6.08	1.56	5.67	1.32	6.99
Acetol	7.23	1.16	4.19	6.07	1.45	5.37	1.18	6.55
Acetone	7.23	1.01	4.12	6.21	1.37	5.18	1.06	6.24
Arabinose	7.53	1.55	4.54	5.98	1.72	6.09	1.55	7.64
Glucose	7.57	1.66	4.61	5.91	1.80	6.28	1.66	7.94
d-Glyceraldehyde	7.63	1.54	4.58	6.09	1.72	6.12	1.54	7.66
Glycolaldehyde	7.58	1.58	4.58	5.99	1.75	6.17	1.59	7.75
Glyoxal	7.75	3.35	5.55	4.40	3.50	10.05	4.50	14.55
l-Glyceraldehyde	7.63	1.54	4.58	6.09	1.72	6.12	1.54	7.66
Methylglyoxal	7.55	3.14	5.34	4.42	3.23	9.41	4.07	13.48
Ribose	7.77	1.68	4.73	6.08	1.83	6.41	1.69	8.10

Table S1B: Descriptors J_I, J_A, J_{HL}, J_χ, J_η, J_ω, J_{D1}, J_{ω^+}, J_{ω^-}, $J_{\Delta\omega^\pm}$ and J_{D2} for the Acetaldehyde, Acetol, Acetone, Arabinose, Glucose, d-Glyceraldehyde, Glyoxal, l-Glyceraldehyde, Methylglyoxal and Ribose molecules calculated from the results of Table S1A

	J_I	J_A	J_{HL}	J_χ	J_η	J_ω	J_{D1}	J_{ω^-}	J_{ω^+}	$J_{\Delta\omega^\pm}$	J_{D2}
Acetaldehyde	2.96	2.92	4.16	0.02	5.88	0.76	5.93	1.14	1.16	2.30	2.82
Acetol	2.75	2.79	3.91	0.02	5.53	0.70	5.58	1.06	1.04	2.10	2.57
Acetone	2.96	2.80	4.07	0.08	5.76	0.63	5.79	0.86	0.94	1.80	2.20
Arabinose	2.59	2.86	3.85	0.14	5.44	0.87	5.51	1.48	1.34	2.82	3.45
Glucose	2.41	2.85	3.73	0.22	5.26	0.94	5.35	1.65	1.43	3.09	3.78
d-Glyceraldehyde	2.85	2.87	4.05	0.01	5.72	0.84	5.78	1.32	1.31	2.63	3.22
Glycolaldehyde	2.90	2.89	4.09	0.00	5.79	0.86	5.85	1.35	1.36	2.71	3.32
Glyoxal	2.58	2.73	3.76	0.07	5.32	1.96	5.67	3.62	3.55	7.17	8.78
l-Glyceraldehyde	2.85	2.87	4.05	0.01	5.72	0.84	5.78	1.32	1.31	2.63	3.22
Methylglyoxal	2.65	2.73	3.80	0.04	5.38	1.80	5.67	3.28	3.24	6.52	7.99
Ribose	2.39	2.84	3.71	0.22	5.23	0.94	5.32	1.66	1.44	3.10	3.80
Average	2.72	2.83	3.93	0.08	5.55	1.01	5.66	1.70	1.65	3.35	4.11

Table S2A: HOMO and LUMO orbital energies (in eV), ionization potentials I and electron affinities A (in eV), and global electronegativity χ, total hardness η, global electrophilicity ω, electrodonating power (ω^-), electroaccepting power (ω^+), and net electrophilicity $\Delta\omega^\pm$ of Acetaldehyde, Acetol, Acetone, Arabinose, Glucose, d-Glyceraldehyde, Glyoxal, l-Glyceraldehyde, Methylglyoxal and Ribose calculated with the M11L density functional and the Def2TZVP basis set using water as solvent simulated with the SMD parametrization of the IEF-PCM model. The upper part of the table shows the results derived assuming the validity of KID procedure and the lower part shows the results derived from the calculated vertical I and A.

Property	HOMO	LUMO	χ_K	η_K	ω_K	ω_K^-	ω_K^+	$\Delta\omega_K^\pm$
Acetaldehyde	-6.65	-1.49	4.07	5.16	1.60	5.56	1.50	7.06
Acetol	-6.33	-1.38	3.86	4.95	1.50	5.24	1.38	6.62
Acetone	-6.55	-1.25	3.90	5.31	1.43	5.15	1.25	6.39
Arabinose	-6.43	-1.72	4.08	4.71	1.77	5.87	1.79	7.65
Glucose	-6.39	-1.86	4.13	4.53	1.88	6.10	1.98	8.08
d-Glyceraldehyde	-6.71	-1.75	4.23	4.96	1.80	6.03	1.80	7.83
Glycolaldehyde	-6.73	-1.79	4.26	4.94	1.84	6.11	1.85	7.96
Glyoxal	-6.59	-3.73	5.16	2.86	4.66	12.07	6.91	18.99
l-Glyceraldehyde	-6.71	-1.75	4.23	4.96	1.80	6.03	1.80	7.83
Methylglyoxal	-6.48	-3.42	4.95	3.06	4.00	10.67	5.72	16.39
Ribose	-6.53	-1.87	4.20	4.66	1.90	6.19	1.98	8.17
Property	I	A	χ	η	ω	ω^-	ω^+	$\Delta\omega^\pm$
Acetaldehyde	7.15	1.21	4.18	5.94	1.47	5.40	1.22	6.62
Acetol	6.82	1.11	3.97	5.71	1.38	5.10	1.13	6.22
Acetone	7.01	0.97	3.99	6.04	1.32	5.01	1.02	6.04
Arabinose	6.82	1.45	4.14	5.37	1.59	5.59	1.45	7.04
Glucose	6.74	1.56	4.15	5.18	1.66	5.72	1.58	7.30
d-Glyceraldehyde	7.16	1.44	4.30	5.72	1.62	5.75	1.44	7.19
Glycolaldehyde	7.26	1.51	4.39	5.75	1.67	5.90	1.51	7.41
Glyoxal	7.16	3.32	5.24	3.84	3.58	10.01	4.77	14.79
l-Glyceraldehyde	7.16	1.44	4.30	5.72	1.62	5.75	1.44	7.19
Methylglyoxal	7.03	3.04	5.03	3.98	3.18	9.12	4.09	13.21
Ribose	6.88	1.59	4.23	5.29	1.69	5.83	1.60	7.43

Table S2B: Descriptors J_I, J_A, J_{HL}, J_χ, J_η, J_ω, J_{D1}, J_{ω^+}, J_{ω^-}, $J_{\Delta\omega^\pm}$ and J_{D2} for the Acetaldehyde, Acetol, Acetone, Arabinose, Glucose, d-Glyceraldehyde, Glyoxal, l-Glyceraldehyde, Methylglyoxal and Ribose molecules calculated from the results of Table S2A

	J_I	J_A	J_{HL}	J_χ	J_η	J_ω	J_{D1}	J_{ω^-}	J_{ω^+}	$J_{\Delta\omega^\pm}$	J_{D2}
Acetaldehyde	0.51	0.28	0.58	0.11	0.79	0.13	0.81	0.16	0.27	0.44	0.54
Acetol	0.49	0.27	0.56	0.11	0.75	0.12	0.77	0.14	0.25	0.40	0.49
Acetone	0.46	0.27	0.54	0.09	0.73	0.11	0.75	0.13	0.23	0.36	0.44
Arabinose	0.39	0.27	0.47	0.06	0.66	0.17	0.69	0.28	0.33	0.61	0.75
Glucose	0.35	0.30	0.46	0.02	0.64	0.22	0.68	0.38	0.40	0.78	0.96
d-Glyceraldehyde	0.45	0.30	0.55	0.07	0.76	0.18	0.78	0.28	0.36	0.64	0.78
Glycolaldehyde	0.53	0.28	0.59	0.13	0.80	0.16	0.83	0.21	0.34	0.55	0.68
Glyoxal	0.57	0.41	0.70	0.08	0.98	1.08	1.46	2.06	2.14	4.20	5.14
l-Glyceraldehyde	0.45	0.30	0.55	0.07	0.76	0.18	0.78	0.28	0.36	0.64	0.78
Methylglyoxal	0.55	0.38	0.67	0.08	0.93	0.82	1.24	1.55	1.63	3.18	3.89
Ribose	0.35	0.29	0.45	0.03	0.64	0.20	0.67	0.35	0.38	0.73	0.90
Average	0.46	0.30	0.56	0.08	0.77	0.31	0.86	0.53	0.61	1.14	1.40

Table S3A: HOMO and LUMO orbital energies (in eV), ionization potentials I and electron affinities A (in eV), and global electronegativity χ, total hardness η, global electrophilicity ω, electrodonating power (ω^-), electroaccepting power (ω^+), and net electrophilicity $\Delta\omega^\pm$ of Acetaldehyde, Acetol, Acetone, Arabinose, Glucose, d-Glyceraldehyde, Glyoxal, l-Glyceraldehyde, Methylglyoxal and Ribose calculated with the MN12L density functional and the Def2TZVP basis set using water as solvent simulated with the SMD parametrization of the IEF-PCM model. The upper part of the table shows the results derived assuming the validity of KID procedure and the lower part shows the results derived from the calculated vertical I and A.

Property	HOMO	LUMO	χ_K	η_K	ω_K	ω_K^-	ω_K^+	$\Delta\omega_K^\pm$
Acetaldehyde	-6.60	-1.15	3.88	5.45	1.38	5.04	1.16	6.20
Acetol	-6.24	-1.06	3.65	5.18	1.29	4.72	1.07	5.79
Acetone	-6.46	-0.93	3.69	5.53	1.23	4.66	0.96	5.62
Arabinose	-6.37	-1.39	3.88	4.97	1.51	5.27	1.40	6.67
Glucose	-6.25	-1.49	3.87	4.76	1.57	5.38	1.51	6.89
d-Glyceraldehyde	-6.71	-1.40	4.05	5.31	1.55	5.45	1.40	6.85
Glycolaldehyde	-6.70	-1.44	4.07	5.26	1.58	5.52	1.45	6.97
Glyoxal	-6.62	-3.44	5.03	3.17	3.99	10.68	5.65	16.34
l-Glyceraldehyde	-6.71	-1.40	4.05	5.31	1.55	5.45	1.40	6.85
Methylglyoxal	-6.49	-3.17	4.83	3.32	3.51	9.65	4.82	14.47
Ribose	-6.40	-1.50	3.95	4.89	1.59	5.47	1.52	6.99
Property	I	A	χ	η	ω	ω^-	ω^+	$\Delta\omega^\pm$
Acetaldehyde	6.96	0.92	3.94	6.04	1.28	4.92	0.98	5.89
Acetol	6.62	0.79	3.70	5.83	1.18	4.57	0.87	5.44
Acetone	6.78	0.65	3.71	6.14	1.12	4.49	0.77	5.26
Arabinose	6.69	1.17	3.93	5.51	1.40	5.11	1.18	6.29
Glucose	6.59	1.25	3.92	5.34	1.44	5.17	1.25	6.43
d-Glyceraldehyde	7.05	1.16	4.10	5.89	1.43	5.28	1.18	6.46
Glycolaldehyde	7.09	1.23	4.16	5.87	1.47	5.40	1.24	6.63
Glyoxal	7.11	3.07	5.09	4.04	3.21	9.22	4.13	13.35
l-Glyceraldehyde	7.05	1.16	4.10	5.89	1.43	5.28	1.18	6.46
Methylglyoxal	6.96	2.83	4.89	4.13	2.90	8.50	3.60	12.10
Ribose	6.73	1.28	4.01	5.45	1.47	5.29	1.28	6.57

Table S3B: Descriptors J_I, J_A, J_{HL}, J_χ, J_η, J_ω, J_{D1}, J_{ω^+}, J_{ω^-}, $J_{\Delta\omega^\pm}$ and J_{D2} for the Acetaldehyde, Acetol, Acetone, Arabinose, Glucose, d-Glyceraldehyde, Glyoxal, l-Glyceraldehyde, Methylglyoxal and Ribose molecules calculated from the results of Table S3A

	J_I	J_A	J_{HL}	J_χ	J_η	J_ω	J_{D1}	J_{ω^-}	J_{ω^+}	$J_{\Delta\omega^\pm}$	J_{D2}
Acetaldehyde	0.36	0.23	0.43	0.06	0.59	0.09	0.60	0.12	0.18	0.30	0.37
Acetol	0.38	0.27	0.47	0.06	0.65	0.11	0.66	0.15	0.20	0.35	0.43
Acetone	0.32	0.28	0.43	0.02	0.60	0.11	0.61	0.17	0.19	0.36	0.44
Arabinose	0.32	0.22	0.39	0.05	0.54	0.11	0.55	0.16	0.21	0.38	0.46
Glucose	0.34	0.24	0.41	0.05	0.58	0.14	0.60	0.21	0.26	0.47	0.58
d-Glyceraldehyde	0.34	0.24	0.42	0.05	0.58	0.12	0.60	0.17	0.22	0.39	0.48
Glycolaldehyde	0.39	0.22	0.45	0.09	0.61	0.10	0.62	0.12	0.21	0.33	0.41
Glyoxal	0.50	0.37	0.62	0.07	0.87	0.77	1.16	1.46	1.53	2.99	3.66
l-Glyceraldehyde	0.34	0.24	0.42	0.05	0.58	0.12	0.60	0.17	0.22	0.39	0.48
Methylglyoxal	0.47	0.34	0.58	0.06	0.81	0.62	1.02	1.15	1.22	2.37	2.90
Ribose	0.34	0.22	0.40	0.06	0.56	0.12	0.57	0.18	0.24	0.42	0.51
Average	0.37	0.26	0.46	0.06	0.63	0.22	0.69	0.37	0.43	0.80	0.98

Table S4A: HOMO and LUMO orbital energies (in eV), ionization potentials I and electron affinities A (in eV), and global electronegativity χ, total hardness η, global electrophilicity ω, electrodonating power (ω^-), electroaccepting power (ω^+), and net electrophilicity $\Delta\omega^\pm$ of Acetaldehyde, Acetol, Acetone, Arabinose, Glucose, d-Glyceraldehyde, Glyoxal, l-Glyceraldehyde, Methylglyoxal and Ribose calculated with the MN12SX density functional and the Def2TZVP basis set using water as solvent simulated with the SMD parametrization of the IEF-PCM model. The upper part of the table shows the results derived assuming the validity of KID procedure and the lower part shows the results derived from the calculated vertical I and A.

Property	HOMO	LUMO	χ_K	η_K	ω_K	ω_K^-	ω_K^+	$\Delta\omega_K^\pm$
Acetaldehyde	-7.52	-0.94	4.23	6.58	1.36	5.25	1.02	6.27
Acetol	-7.14	-0.90	4.02	6.24	1.29	4.98	0.97	5.95
Acetone	-7.37	-0.75	4.06	6.63	1.24	4.93	0.87	5.81
Arabinose	-7.25	-1.20	4.22	6.06	1.47	5.44	1.21	6.65
Glucose	-7.14	-1.29	4.21	5.85	1.52	5.50	1.29	6.80
d-Glyceraldehyde	-7.62	-1.19	4.40	6.43	1.51	5.62	1.22	6.84
Glycolaldehyde	-7.61	-1.25	4.43	6.36	1.54	5.70	1.27	6.97
Glyoxal	-7.51	-3.14	5.32	4.37	3.24	9.41	4.09	13.50
l-Glyceraldehyde	-7.62	-1.19	4.40	6.43	1.51	5.62	1.22	6.84
Methylglyoxal	-7.37	-2.90	5.13	4.47	2.95	8.74	3.61	12.34
Ribose	-7.28	-1.32	4.30	5.95	1.55	5.63	1.33	6.96
Property	I	A	χ	η	ω	ω^-	ω^+	$\Delta\omega^\pm$
Acetaldehyde	7.21	1.17	4.19	6.04	1.45	5.37	1.19	6.56
Acetol	6.96	1.06	4.01	5.90	1.36	5.09	1.09	6.18
Acetone	7.04	0.92	3.98	6.12	1.29	4.96	0.98	5.93
Arabinose	7.17	1.40	4.29	5.78	1.59	5.68	1.40	7.08
Glucose	7.09	1.49	4.29	5.61	1.64	5.78	1.49	7.26
d-Glyceraldehyde	7.37	1.39	4.38	5.98	1.60	5.77	1.39	7.17
Glycolaldehyde	7.35	1.45	4.40	5.89	1.64	5.85	1.45	7.31
Glyoxal	7.46	3.24	5.35	4.22	3.40	9.73	4.38	14.11
l-Glyceraldehyde	7.37	1.39	4.38	5.98	1.60	5.77	1.39	7.17
Methylglyoxal	7.29	3.01	5.15	4.28	3.10	9.04	3.89	12.93
Ribose	7.23	1.52	4.38	5.72	1.67	5.89	1.52	7.41

Table S4B: Descriptors J_I, J_A, J_{HL}, J_χ, J_η, J_ω, J_{D1}, J_{ω^+}, J_{ω^-}, $J_{\Delta\omega^\pm}$ and J_{D2} for the Acetaldehyde, Acetol, Acetone, Arabinose, Glucose, d-Glyceraldehyde, Glyoxal, l-Glyceraldehyde, Methylglyoxal and Ribose molecules calculated from the results of Table S4A

	J_I	J_A	J_{HL}	J_χ	J_η	J_ω	J_{D1}	J_{ω^-}	J_{ω^+}	$J_{\Delta\omega^\pm}$	J_{D2}
Acetaldehyde	0.32	0.22	0.39	0.05	0.54	0.09	0.55	0.12	0.17	0.29	0.36
Acetol	0.18	0.16	0.24	0.01	0.35	0.07	0.35	0.11	0.12	0.23	0.28
Acetone	0.33	0.17	0.37	0.08	0.50	0.05	0.51	0.02	0.11	0.13	0.17
Arabinose	0.08	0.20	0.22	0.06	0.28	0.12	0.31	0.25	0.19	0.43	0.53
Glucose	0.04	0.20	0.20	0.08	0.24	0.12	0.28	0.27	0.19	0.47	0.57
d-Glyceraldehyde	0.24	0.20	0.31	0.02	0.44	0.10	0.45	0.15	0.17	0.33	0.40
Glycolaldehyde	0.27	0.20	0.33	0.03	0.47	0.10	0.48	0.15	0.18	0.34	0.41
Glyoxal	0.04	0.11	0.12	0.03	0.15	0.16	0.22	0.32	0.29	0.61	0.75
l-Glyceraldehyde	0.24	0.20	0.31	0.02	0.44	0.10	0.45	0.15	0.17	0.33	0.40
Methylglyoxal	0.07	0.11	0.13	0.02	0.18	0.15	0.24	0.31	0.28	0.59	0.72
Ribose	0.04	0.19	0.20	0.07	0.24	0.12	0.28	0.26	0.19	0.45	0.56
Average	0.17	0.18	0.26	0.04	0.35	0.11	0.37	0.19	0.19	0.38	0.47

Table S5A: HOMO and LUMO orbital energies (in eV), ionization potentials I and electron affinities A (in eV), and global electronegativity χ, total hardness η, global electrophilicity ω, electrodonating power (ω^-), electroaccepting power (ω^+), and net electrophilicity $\Delta\omega^\pm$ of Acetaldehyde, Acetol, Acetone, Arabinose, Glucose, d-Glyceraldehyde, Glyoxal, l-Glyceraldehyde, Methylglyoxal and Ribose calculated with the N12 density functional and the Def2TZVP basis set using water as solvent simulated with the SMD parametrization of the IEF-PCM model. The upper part of the table shows the results derived assuming the validity of KID procedure and the lower part shows the results derived from the calculated vertical I and A.

Property	HOMO	LUMO	χ_K	η_K	ω_K	ω_K^-	ω_K^+	$\Delta\omega_K^\pm$
Acetaldehyde	-6.01	-1.66	3.84	4.34	1.69	5.58	1.74	7.32
Acetol	-5.70	-1.52	3.61	4.17	1.56	5.19	1.58	6.77
Acetone	-5.84	-1.40	3.62	4.44	1.47	5.03	1.41	6.44
Arabinose	-5.87	-1.87	3.87	4.00	1.87	5.94	2.06	8.00
Glucose	-5.76	-2.07	3.91	3.70	2.07	6.33	2.42	8.75
d-Glyceraldehyde	-6.10	-1.88	3.99	4.22	1.89	6.03	2.04	8.08
Glycolaldehyde	-6.14	-1.93	4.03	4.21	1.93	6.14	2.11	8.25
Glyoxal	-6.12	-3.89	5.01	2.23	5.62	13.89	8.88	22.77
l-Glyceraldehyde	-6.10	-1.88	3.99	4.22	1.89	6.03	2.04	8.08
Methylglyoxal	-5.96	-3.57	4.77	2.39	4.76	12.05	7.28	19.33
Ribose	-5.95	-2.09	4.02	3.86	2.09	6.43	2.41	8.84
Property	I	A	χ	η	ω	ω^-	ω^+	$\Delta\omega^\pm$
Acetaldehyde	6.90	0.99	3.95	5.91	1.32	4.97	1.03	6.00
Acetol	6.38	0.88	3.63	5.50	1.20	4.55	0.92	5.47
Acetone	6.69	0.74	3.71	5.95	1.16	4.55	0.83	5.38
Arabinose	6.31	1.20	3.76	5.11	1.38	4.96	1.20	6.16
Glucose	6.16	1.37	3.76	4.79	1.48	5.14	1.38	6.51
d-Glyceraldehyde	6.71	1.20	3.95	5.52	1.42	5.16	1.20	6.36
Glycolaldehyde	6.94	1.27	4.11	5.66	1.49	5.38	1.28	6.66
Glyoxal	6.87	3.23	5.05	3.64	3.50	9.76	4.71	14.47
l-Glyceraldehyde	6.71	1.20	3.95	5.52	1.42	5.16	1.20	6.36
Methylglyoxal	6.66	2.94	4.80	3.72	3.10	8.84	4.03	12.87
Ribose	6.35	1.41	3.88	4.94	1.52	5.29	1.41	6.71

Table S5B: Descriptors J_I, J_A, J_{HL}, J_χ, J_η, J_ω, J_{D1}, J_{ω^+}, J_{ω^-}, $J_{\Delta\omega^\pm}$ and J_{D2} for the Acetaldehyde, Acetol, Acetone, Arabinose, Glucose, d-Glyceraldehyde, Glyoxal, l-Glyceraldehyde, Methylglyoxal and Ribose molecules calculated from the results of Table S5A

	J_I	J_A	J_{HL}	J_χ	J_η	J_ω	J_{D1}	J_{ω^-}	J_{ω^+}	$J_{\Delta\omega^\pm}$	J_{D2}
Acetaldehyde	0.90	0.68	1.12	0.11	1.57	0.38	1.62	0.60	0.71	1.32	1.61
Acetol	0.68	0.65	0.94	0.02	1.32	0.37	1.37	0.64	0.66	1.30	1.59
Acetone	0.86	0.66	1.08	0.10	1.51	0.31	1.55	0.48	0.58	1.06	1.31
Arabinose	0.44	0.67	0.80	0.12	1.11	0.49	1.22	0.98	0.86	1.84	2.25
Glucose	0.40	0.70	0.80	0.15	1.09	0.59	1.25	1.19	1.04	2.24	2.74
d-Glyceraldehyde	0.61	0.69	0.92	0.04	1.30	0.47	1.38	0.88	0.84	1.72	2.11
Glycolaldehyde	0.80	0.65	1.03	0.07	1.45	0.44	1.52	0.76	0.83	1.59	1.95
Glyoxal	0.74	0.66	1.00	0.04	1.41	2.12	2.55	4.13	4.17	8.31	10.17
l-Glyceraldehyde	0.61	0.69	0.92	0.04	1.30	0.47	1.38	0.88	0.84	1.72	2.11
Methylglyoxal	0.70	0.63	0.94	0.04	1.33	1.66	2.13	3.21	3.25	6.47	7.92
Ribose	0.40	0.68	0.79	0.14	1.08	0.57	1.23	1.13	1.00	2.13	2.61
Average	0.65	0.67	0.94	0.08	1.32	0.72	1.56	1.35	1.34	2.70	3.31

Table S6A: HOMO and LUMO orbital energies (in eV), ionization potentials I and electron affinities A (in eV), and global electronegativity χ, total hardness η, global electrophilicity ω, electrodonating power (ω^-), electroaccepting power (ω^+), and net electrophilicity $\Delta\omega^{\pm}$ of Acetaldehyde, Acetol, Acetone, Arabinose, Glucose, d-Glyceraldehyde, Glyoxal, l-Glyceraldehyde, Methylglyoxal and Ribose calculated with the N12SX density functional and the Def2TZVP basis set using water as solvent simulated with the SMD parametrization of the IEF-PCM model. The upper part of the table shows the results derived assuming the validity of KID procedure and the lower part shows the results derived from the calculated vertical I and A.

Property	HOMO	LUMO	χ_K	η_K	ω_K	ω_K^-	ω_K^+	$\Delta\omega_K^{\pm}$
Acetaldehyde	-7.21	-0.98	4.10	6.23	1.35	5.13	1.03	6.16
Acetol	-6.88	-0.89	3.88	5.99	1.26	4.83	0.95	5.78
Acetone	-7.05	-0.73	3.89	6.31	1.20	4.74	0.85	5.59
Arabinose	-7.04	-1.22	4.13	5.82	1.47	5.36	1.23	6.60
Glucose	-6.91	-1.36	4.14	5.55	1.54	5.50	1.36	6.86
d-Glyceraldehyde	-7.33	-1.22	4.28	6.11	1.50	5.52	1.24	6.75
Glycolaldehyde	-7.34	-1.27	4.30	6.08	1.52	5.58	1.28	6.86
Glyoxal	-7.31	-3.27	5.29	4.04	3.46	9.81	4.53	14.34
l-Glyceraldehyde	-7.33	-1.22	4.28	6.11	1.50	5.52	1.24	6.75
Methylglyoxal	-7.15	-3.00	5.07	4.15	3.10	9.00	3.93	12.93
Ribose	-7.09	-1.40	4.25	5.70	1.58	5.64	1.40	7.04
Property	I	A	χ	η	ω	ω^-	ω^+	$\Delta\omega^{\pm}$
Acetaldehyde	7.12	1.16	4.14	5.96	1.44	5.32	1.18	6.50
Acetol	6.83	1.03	3.93	5.81	1.33	4.98	1.06	6.04
Acetone	6.93	0.88	3.90	6.05	1.26	4.84	0.94	5.79
Arabinose	7.03	1.37	4.20	5.66	1.56	5.57	1.37	6.94
Glucose	6.91	1.50	4.20	5.40	1.64	5.71	1.51	7.22
d-Glyceraldehyde	7.28	1.37	4.33	5.91	1.58	5.70	1.37	7.07
Glycolaldehyde	7.28	1.44	4.36	5.83	1.63	5.80	1.44	7.24
Glyoxal	7.37	3.33	5.35	4.04	3.54	10.0	14.67	14.68
l-Glyceraldehyde	7.28	1.37	4.33	5.91	1.58	5.70	1.37	7.07
Methylglyoxal	7.17	3.08	5.12	4.10	3.20	9.23	4.10	13.33
Ribose	7.09	1.54	4.31	5.56	1.68	5.86	1.54	7.40

Table S6B: Descriptors J_I, J_A, J_{HL}, J_χ, J_η, J_ω, J_{D1}, J_{ω^+}, J_{ω^-}, $J_{\Delta\omega^\pm}$ and J_{D2} for the Acetaldehyde, Acetol, Acetone, Arabinose, Glucose, d-Glyceraldehyde, Glyoxal, l-Glyceraldehyde, Methylglyoxal and Ribose molecules calculated from the results of Table S6A

	J_I	J_A	J_{HL}	J_χ	J_η	J_ω	J_{D1}	J_{ω^-}	J_{ω^+}	$J_{\Delta\omega^\pm}$	J_{D2}
Acetaldehyde	0.09	0.18	0.20	0.04	0.28	0.09	0.29	0.19	0.15	0.33	0.41
Acetol	0.05	0.14	0.15	0.05	0.19	0.07	0.20	0.15	0.11	0.26	0.32
Acetone	0.12	0.14	0.18	0.01	0.26	0.06	0.27	0.11	0.10	0.20	0.25
Arabinose	0.01	0.15	0.15	0.07	0.16	0.09	0.20	0.21	0.14	0.35	0.43
Glucose	0.01	0.14	0.14	0.07	0.15	0.09	0.19	0.21	0.15	0.36	0.44
d-Glyceraldehyde	0.05	0.15	0.16	0.05	0.20	0.09	0.22	0.18	0.14	0.32	0.39
Glycolaldehyde	0.07	0.18	0.19	0.05	0.24	0.10	0.27	0.22	0.16	0.38	0.47
Glyoxal	0.06	0.06	0.09	0.06	0.00	0.08	0.10	0.20	0.14	0.34	0.42
l-Glyceraldehyde	0.05	0.15	0.16	0.05	0.20	0.09	0.22	0.18	0.14	0.32	0.39
Methylglyoxal	0.02	0.08	0.08	0.05	0.06	0.10	0.13	0.23	0.18	0.40	0.49
Ribose	0.00	0.14	0.14	0.07	0.14	0.09	0.18	0.21	0.14	0.36	0.44
Average	0.05	0.14	0.15	0.05	0.17	0.09	0.21	0.19	0.14	0.33	0.41

Table S7A: HOMO and LUMO orbital energies (in eV), ionization potentials I and electron affinities A (in eV), and global electronegativity χ, total hardness η, global electrophilicity ω, electrodonating power (ω^-), electroaccepting power (ω^+), and net electrophilicity $\Delta\omega^\pm$ of Acetaldehyde, Acetol, Acetone, Arabinose, Glucose, d-Glyceraldehyde, Glyoxal, l-Glyceraldehyde, Methylglyoxal and Ribose calculated with the SOGGA11 density functional and the Def2TZVP basis set using water as solvent simulated with the SMD parametrization of the IEF-PCM model. The upper part of the table shows the results derived assuming the validity of KID procedure and the lower part shows the results derived from the calculated vertical I and A.

Property	HOMO	LUMO	χ_K	η_K	ω_K	ω_K^-	ω_K^+	$\Delta\omega_K^\pm$
Acetaldehyde	-5.98	-1.86	3.92	4.12	1.87	5.95	2.03	7.99
Acetol	-5.68	-1.73	3.70	3.95	1.74	5.57	1.87	7.44
Acetone	-5.83	-1.62	3.73	4.21	1.65	5.42	1.70	7.12
Arabinose	-5.87	-2.16	4.02	3.71	2.18	6.60	2.58	9.17
Glucose	-5.89	-2.33	4.11	3.56	2.38	7.03	2.92	9.95
d-Glyceraldehyde	-6.03	-2.20	4.12	3.83	2.21	6.72	2.60	9.32
Glycolaldehyde	-6.13	-2.13	4.13	4.00	2.13	6.58	2.45	9.03
Glyoxal	-6.09	-4.09	5.09	2.00	6.48	15.63	10.54	26.17
l-Glyceraldehyde	-6.03	-2.20	4.12	3.83	2.21	6.72	2.60	9.32
Methylglyoxal	-5.91	-3.75	4.83	2.15	5.42	13.39	8.56	21.96
Ribose	-5.79	-2.14	3.96	3.65	2.15	6.51	2.54	9.05
Property	I	A	χ	η	ω	ω^-	ω^+	$\Delta\omega^\pm$
Acetaldehyde	7.06	1.21	4.13	5.85	1.46	5.35	1.22	6.57
Acetol	6.47	1.09	3.78	5.37	1.33	4.89	1.11	5.99
Acetone	6.86	-3.48	1.69	10.34	0.14	1.76	0.08	1.84
Arabinose	6.29	1.39	3.84	4.90	1.50	5.23	1.40	6.63
Glucose	6.28	1.52	3.90	4.76	1.60	5.44	1.54	6.98
d-Glyceraldehyde	6.59	1.41	4.00	5.18	1.54	5.41	1.41	6.82
Glycolaldehyde	7.03	1.47	4.25	5.56	1.62	5.72	1.47	7.19
Glyoxal	6.96	3.42	5.19	3.53	3.81	10.44	5.25	15.69
l-Glyceraldehyde	6.59	1.41	4.00	5.18	1.54	5.41	1.41	6.82
Methylglyoxal	6.72	3.12	4.92	3.60	3.37	9.42	4.50	13.91
Ribose	6.32	1.37	3.85	4.95	1.50	5.23	1.38	6.61

Table S7B: Descriptors J_I, J_A, J_{HL}, J_χ, J_η, J_ω, J_{D1}, J_{ω^+}, J_{ω^-}, $J_{\Delta\omega^\pm}$ and J_{D2} for the Acetaldehyde, Acetol, Acetone, Arabinose, Glucose, d-Glyceraldehyde, Glyoxal, l-Glyceraldehyde, Methylglyoxal and Ribose molecules calculated from the results of Table S7A

	J_I	J_A	J_{HL}	J_χ	J_η	J_ω	J_{D1}	J_{ω^-}	J_{ω^+}	$J_{\Delta\omega^\pm}$	J_{D2}
Acetaldehyde	1.07	0.65	1.26	0.21	1.73	0.41	1.79	0.60	0.81	1.41	1.74
Acetol	0.79	0.63	1.01	0.08	1.42	0.41	1.48	0.68	0.76	1.44	1.77
Acetone	1.02	5.10	5.20	2.04	6.12	1.51	6.63	3.66	1.62	5.27	6.62
Arabinose	0.42	0.77	0.88	0.18	1.19	0.67	1.38	1.36	1.18	2.54	3.12
Glucose	0.39	0.81	0.90	0.21	1.20	0.78	1.45	1.59	1.38	2.97	3.64
d-Glyceraldehyde	0.55	0.79	0.97	0.12	1.34	0.67	1.51	1.31	1.19	2.50	3.07
Glycolaldehyde	0.91	0.66	1.12	0.12	1.57	0.51	1.65	0.86	0.98	1.83	2.25
Glyoxal	0.87	0.67	1.09	0.10	1.53	2.67	3.08	5.19	5.29	10.48	12.84
l-Glyceraldehyde	0.55	0.79	0.97	0.12	1.34	0.67	1.51	1.31	1.19	2.50	3.07
Methylglyoxal	0.81	0.63	1.03	0.09	1.45	2.06	2.52	3.98	4.07	8.05	9.85
Ribose	0.53	0.76	0.93	0.11	1.29	0.65	1.45	1.28	1.16	2.44	3.00
Average	0.72	1.12	1.40	0.31	1.84	1.00	2.22	1.98	1.79	3.77	4.63

Table S8A: HOMO and LUMO orbital energies (in eV), ionization potentials I and electron affinities A (in eV), and global electronegativity χ, total hardness η, global electrophilicity ω, electrodonating power (ω^-), electroaccepting power (ω^+), and net electrophilicity $\Delta\omega^\pm$ of Acetaldehyde, Acetol, Acetone, Arabinose, Glucose, d-Glyceraldehyde, Glyoxal, l-Glyceraldehyde, Methylglyoxal and Ribose calculated with the SOGGA11X density functional and the Def2TZVP basis set using water as solvent simulated with the SMD parametrization of the IEF-PCM model. The upper part of the table shows the results derived assuming the validity of KID procedure and the lower part shows the results derived from the calculated vertical I and A.

Property	HOMO	LUMO	χ_K	η_K	ω_K	ω_K^-	ω_K^+	$\Delta\omega_K^\pm$
Acetaldehyde	-8.65	0.09	4.28	8.74	1.05	4.78	0.50	5.28
Acetol	-8.28	0.14	4.07	8.42	0.98	4.52	0.46	4.98
Acetone	-8.48	0.31	4.08	8.79	0.95	4.49	0.40	4.89
Arabinose	-8.69	-0.42	4.56	8.27	1.25	5.30	0.75	6.05
Glucose	-8.29	-0.40	4.34	7.90	1.19	5.05	0.71	5.77
d-Glyceraldehyde	-8.76	-0.17	4.46	8.59	1.16	5.09	0.62	5.71
Glycolaldehyde	-8.76	-0.21	4.49	8.55	1.18	5.13	0.64	5.78
Glyoxal	-8.67	-2.17	5.42	6.49	2.26	7.64	2.22	9.86
l-Glyceraldehyde	-8.76	-0.17	4.46	8.59	1.16	5.09	0.62	5.71
Methylglyoxal	-8.52	-1.95	5.23	6.57	2.08	7.20	1.96	9.16
Ribose	-8.30	-0.08	4.19	8.22	1.07	4.75	0.56	5.31
Property	I	A	χ	η	ω	ω^-	ω^+	$\Delta\omega^\pm$
Acetaldehyde	7.23	1.19	4.21	6.04	1.47	5.42	1.21	6.63
Acetol	7.04	1.04	4.04	6.00	1.36	5.12	1.08	6.19
Acetone	7.06	0.88	3.97	6.17	1.28	4.92	0.95	5.88
Arabinose	7.47	1.62	4.55	5.84	1.77	6.18	1.63	7.80
Glucose	7.35	1.65	4.50	5.71	1.77	6.15	1.65	7.81
d-Glyceraldehyde	7.43	1.41	4.42	6.02	1.63	5.84	1.42	7.25
Glycolaldehyde	7.40	1.48	4.44	5.92	1.66	5.92	1.48	7.40
Glyoxal	7.61	3.31	5.46	4.30	3.46	9.92	4.46	14.38
l-Glyceraldehyde	7.43	1.41	4.42	6.02	1.63	5.84	1.42	7.25
Methylglyoxal	7.42	3.07	5.24	4.35	3.16	9.21	3.97	13.18
Ribose	7.13	1.28	4.21	5.85	1.51	5.50	1.29	6.79

Table S8B: Descriptors J_I, J_A, J_{HL}, J_χ, J_η, J_ω, J_{D1}, J_{ω^+}, J_{ω^-}, $J_{\Delta\omega^\pm}$ and J_{D2} for the Acetaldehyde, Acetol, Acetone, Arabinose, Glucose, d-Glyceraldehyde, Glyoxal, l-Glyceraldehyde, Methylglyoxal and Ribose molecules calculated from the results of Table S8A

	J_I	J_A	J_{HL}	J_χ	J_η	J_ω	J_{D1}	J_{ω^-}	J_{ω^+}	$J_{\Delta\omega^\pm}$	J_{D2}
Acetaldehyde	1.42	1.29	1.91	0.07	2.70	0.42	2.74	0.64	0.71	1.35	1.66
Acetol	1.24	1.18	1.71	0.03	2.42	0.38	2.45	0.59	0.62	1.21	1.48
Acetone	1.42	1.20	1.86	0.11	2.62	0.33	2.64	0.44	0.55	0.99	1.21
Arabinose	1.23	1.21	1.72	0.01	2.43	0.51	2.49	0.87	0.88	1.75	2.15
Glucose	0.94	1.25	1.56	0.16	2.19	0.58	2.27	1.10	0.94	2.04	2.50
d-Glyceraldehyde	1.33	1.25	1.82	0.04	2.58	0.47	2.62	0.75	0.79	1.55	1.89
Glycolaldehyde	1.37	1.27	1.87	0.05	2.64	0.49	2.68	0.78	0.83	1.62	1.98
Glyoxal	1.06	1.13	1.55	0.04	2.19	1.20	2.50	2.28	2.24	4.52	5.54
l-Glyceraldehyde	1.33	1.25	1.82	0.04	2.58	0.47	2.62	0.75	0.79	1.55	1.89
Methylglyoxal	1.10	1.12	1.57	0.01	2.22	1.07	2.46	2.02	2.00	4.02	4.92
Ribose	1.17	1.20	1.68	0.01	2.37	0.44	2.41	0.75	0.73	1.48	1.81
Average	1.24	1.21	1.73	0.06	2.45	0.58	2.53	1.00	1.01	2.01	2.46
